# Autophagy in pulmonary fibrosis: friend or foe?

**DOI:** 10.1016/j.gendis.2021.09.008

**Published:** 2021-10-23

**Authors:** Charlotte Hill, Yihua Wang

**Affiliations:** a Biological Sciences, Faculty of Environmental and Life Sciences, University of Southampton, Southampton SO17 1BJ, UK; b Institute for Life Sciences, University of Southampton, Southampton SO17 1BJ, UK

**Keywords:** Ageing, Autophagy, EMT, Fibrosis, IPF

## Abstract

Autophagy is an evolutionarily conserved process where long-lived and damaged organelles are degraded. Autophagy has been widely associated with several ageing-process as well in diseases such as neurodegeneration, cancer and fibrosis, and is now being utilised as a target in these diseases. Idiopathic pulmonary fibrosis (IPF) is a progressive, interstitial lung disease with limited treatment options available. It is characterised by abnormal extracellular matrix (ECM) deposition by activated myofibroblasts. It is understood that repetitive micro-injuries to aged-alveolar epithelium combined with genetic factors drive the disease. Several groups have demonstrated that autophagy is altered in IPF although whether autophagy has a protective effect or not is yet to be determined. Autophagy has also been shown to influence many other processes including epithelial-mesenchymal transition (EMT) and endothelial-mesenchymal transition (EndMT) which are known to be key in the pathogenesis of IPF. In this review, we summarise the findings of evidence of altered autophagy in IPF lungs, as well as examine its roles within lung fibrosis. Given these findings, together with the growing use of autophagy manipulation in a clinical setting, this is an exciting area for further research in the study of lung fibrosis.

## Introduction

### Pulmonary fibrosis

Pulmonary fibrosis (PF) is a chronic, interstitial fibrosing lung disease where the thickening and scarring of lung tissue results in increased lung stiffness and reduced gas exchange.^1^, ^2^, ^3^ PF is characterised by aberrant extracellular matrix (ECM) deposition which results in reduced respiratory compliance and ultimately death. It is thought that micro-injuries to aged-lungs lead to an aberrant wound-healing response.^1^^,^^2^^,^^4^ When the cause of PF cannot be identified the disease is termed Idiopathic Pulmonary Fibrosis (IPF).

Reported incidence of IPF vary globally, but generally appear to be rising^5^; the global incidence is estimated to be 10.7 per 100,000 in males and 7.4 per 100,000 in females.^6^ The median survival for patients is only 2–3 years^7^ and currently there are only two approved therapies for IPF; these can only prolong life, so identifying new targets and developing new treatments is crucial.^2^ Patients typically are diagnosed over the age of 55 years old^8^ and more males are diagnosed than females.^9^

The exact causes of IPF are unknown, however, several risk factors for the disease have been identified including environmental factors,^10^, ^11^, ^12^, ^13^ smoking^14^, ^15^, ^16^, and microbial infection.^17^, ^18^, ^19^, ^20^ As described, IPF is an ageing-associated disease and several ‘hallmarks of ageing’ have been identified.^21^, ^22^, ^23^, ^24^, ^25^, ^26^ Autophagy has been found to be dysregulated during ageing; as such, autophagy and ageing have been studied in numerous diseases including IPF.^27^

### Autophagy

**Figure 1:**
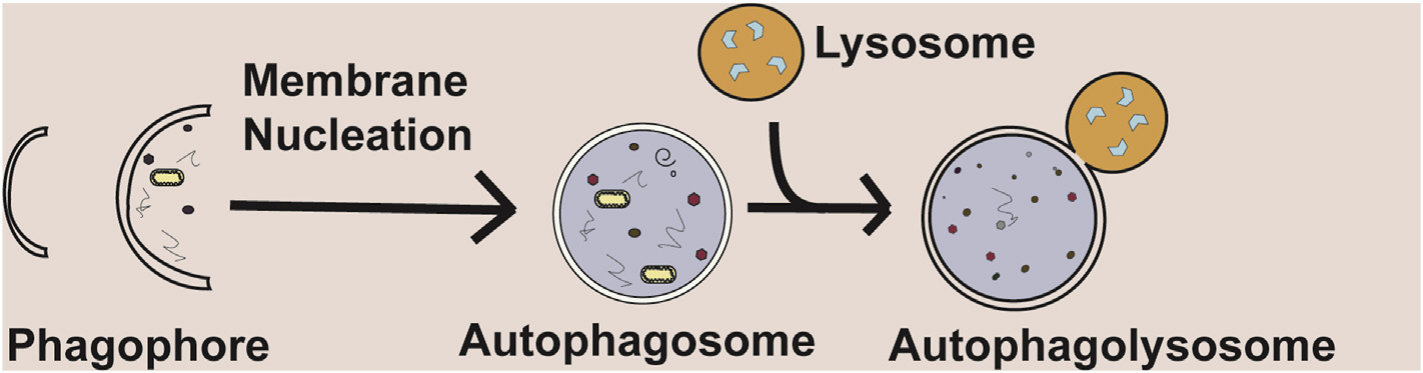
A summary of the process of autophagy. A phagophore is formed, this matures into a double-membraned autophagosome. It fuses with, the enzyme-containing, lysosome, which in turn degrades sequestered material. Adapted from Hill and Wang (2019).^112^

Autophagy (macroautophagy) is a tightly regulated process allowing the bulk or selective degradation of intracellular components, including soluble proteins, aggregated proteins, organelles, foreign bodies and macromolecular complexes.^28^ It is an evolutionarily conserved process occurring ubiquitously in all eukaryotic cells. Autophagy is a dynamic process, capable of responding to stress subsequently limiting cellular damage.^29^ Broadly, autophagy involves the formation of a double-membraned structure which contains material to be degraded, the autophagosome fuses with the lysosome, which in turn degrades the engulfed material^30^ (Fig. 1). The formation of autophagosomes is mainly regulated by autophagy-related (Atg) proteins, of which over 30 have now been identified in yeast.^31^^,^^32^

The association between ageing and autophagy is well-studied,^33^ and in many diseases such as cancer and neurodegeneration, decreased autophagy has also been linked to ageing. Altered autophagy has been reported in several other lung conditions such as asthma^34^ and COPD,^35^ but these diseases show increased levels of autophagy. Recent studies have shown that autophagy may play some roles in fibrogenesis within tissue remodeling and repair. Some studies have shown that autophagy can promote fibrosis,^36^ whilst others have shown reduced autophagy in diseases associated with fibrosis.^37^^,^^38^ Similar findings have also been observed in fibrotic tissue in other organs, such as the kidneys, heart and liver.^39^, ^40^, ^41^, ^42^

Gaining a greater understanding of the role of autophagy in fibrosis in lung disease and elucidating the underlying mechanisms involved will be crucial for developing better treatment strategies. Currently, there are only two approved drugs for the treatment of IPF. Current research suggests that these may alter autophagy to exert their effect but their exact mechanisms are unknown. A better understanding of the pathogenesis of this disease, as well as the drugs which are currently approved, will give hope for future therapies in lung fibrosis, as well as a number of similar conditions.

## Evidence of altered autophagy in fibrotic lungs

A number of studies have evaluated whether autophagy is dysregulated in IPF, a summary of these findings are presented in Table 1. Autophagy was shown to be reduced, Beclin-1 in the cytoplasm of ATII cells in normal regions of IPF lungs had increased expression compared to epithelial cells in other lesions. In the normal ATII cells, Beclin-1 expression was found to be high.^37^ Conversely, Beclin-1 was found to be decreased in IPF by IHC and primary fibroblasts.^43^

**Table 1:** Evidence of altered autophagy in IPF.

Protein	Change	Model	Reference
**p62**	Increased (protein) in IPF *vs.* control.	IHC of IPF *vs.* normal lung	Hill et al (2019)^44^
	Increased in IPF *vs.* control.	IF of IPF *vs.* Control	Patel et al (2012)^38^
	• Only ATII cells express p62 in normal lung. In IPF lungs, p62 was also expressed in ATII cells in ‘normal’ areas without distortion. • p62 was strongly expressed by metaplastic epithelial cells (including cuboidal squamous and bronchiolar-type cells) These cells had some distortion. In areas of dense fibrosis with remodeling, lower expression was observed in honeycomb regions in sub-epithelial fibroblasts. • Staining was also demonstrated in sub-epithelial fibroblasts.	IHC IPF *vs.* normal lung	Araya et al (2013)^37^
**Ubiquitin**	• No expression detected in normal lungs. • ATII cells of ‘normal’ areas of IPF lungs have some staining. • In areas of fibrosis, ubiquitin staining correlated with p62 staining in both ATII cells and fibroblasts. • In honeycomb regions, epithelial and fibroblasts expressed ubiquitinated proteins.	IHC IPF *vs.* normal lung	Araya et al (2013)^37^
	Autophagosomes (visualised by EM): reduced compared to both control and COPD patients.	Electron microscopy	Patel et al (2012)^38^
**LC3**	Dot-like staining of LC3 (resembles autophagosomes) found in ATII cells cytoplasm in the ‘normal’ regions of IPF lung without distortion. However, not in ATII cells of normal lung (confirmed by co-staining for Prosurfactant Protein C). No further staining observed, irrespective of the degree of the fibrosis.	Human IPF tissue	Araya et al (2013)^37^
	LC3B reduced in IPF compared to adjacent normal tissue.	Human IPF tissue	Wang et al (2018)^47^
	LC3-II reduced in IPF *vs.* transplant patients without IPF.	Whole lung homogenate	Patel et al (2012)^38^
**Beclin-1**	Highly diffuse cytoplasmic staining in normal and IPF lung. Cytoplasm of ATII cells in normal areas of IPF lungs had higher expression than epithelial cells in other lesions.	IHC IPF *vs.* normal lung	Araya et al (2013)^37^
	Decreased in IPF.	IHC human tissue and primary fibroblasts	Ricci et al (2013)^43^
**Atg4b**	• Not detectable in healthy tissue. • In IPF lungs, mainly localised to ATII cells typically overlapping FF and hyperplastic epithelial cells, non-ciliated columnar cells and bronchiolar epithelial cells. • Some staining in a few interstitial inflammatory cells was observed. • No staining in fibroblasts.	Human IPF tissue	Cabrera *et al* (2015)^48^
**p-S6**	Upregulated in IPF tissue.	IPF lung tissue compared to healthy lung tissue	Gui et al (2015)^49^
**pAMPK**	Increased in IPF *vs*. control.	Immunoblot IPF lungs	Patel et al (2012)^38^

Our lab recently reported increased levels of p62/SQSTM1 in IPF lungs compared to control, as determined by IHC. We found strong staining in epithelial cells of the thickened alveoli septae in IPF cells; this was found at sites of collagen deposition and fibroblast foci. In the control tissue, there was only weak staining and little collagen deposition.^44^ Other labs have also previously evaluated the levels of p62/SQSTM1 and found it to be increased. It was shown to be only expressed in the ATII cells of normal lung. In IPF lungs, the expression of p62/SQSTM1 was higher in ATII cells in ‘normal’ regions, strong expression was detected in metaplastic epithelial cells. Some staining was observed in sub-epithelial fibroblasts.^37^ Similar findings found p62 of whole lung homogenate, in addition to immunofluorescent (IF) microscopy of p62/aggregate together with electron microscopy to determine the presence of autophagosomes.^38^ Ubiquitin expression was not detected in normal lungs,^37^ ATII cells in normal regions of IPF lungs had low levels of ubiquitin.^37^ In fibrotic regions, ubiquitin staining correlated with p62 staining in ATII cells and fibroblasts. Ubiquitin was observed in both epithelial cells and fibroblasts in honeycomb regions.^37^ Our lab also utilised a publicly available microarray dataset^45^ to evaluate the mRNA levels of *SQSTM1* (p62) in IPF alveolar epithelial cells compared to control, to confirm our findings were as a result of active autophagy. We found that *SQSTM1* (p62) levels were reduced, whilst protein p62 (SQSTM1), evaluated by IHC increased, given that p62 (SQSTM1) is mainly regulated by autophagy,^46^ suggesting autophagy activity was reduced in IPF epithelial cells.^44^

LC3 staining was observed only in ATII cells cytoplasm in normal areas of IPF lung with no distortion; but not in the normal lung. No further staining was observed in any other cell types.^37^ Similar results were demonstrated in IHC of IPF lung tissue which shows reduced LC3B compared to adjacent normal tissue.^47^ LC3-II expression in IPF whole lung homogenate was decreased compared to control lung tissue.^38^ Further, p-AMPK was shown to be increased in IPF compared to control by immunoblot.^38^

Protein expression of Atg4b was shown to be increased in IPF compared to healthy lung tissue. Staining was mainly observed in ATII cells and no staining was observed in fibroblasts.^48^ Phosphorylated-S6 (p-S6) which can be used to determine activation of mTOR and was found p-S6 to be increased in IPF. BLM-treated mice demonstrated elevated p-S6 compared to control. Similarly, IHC of IPF lung tissue showed elevated p-S6.^49^

In general, in human IPF samples it appears autophagy is reduced. However, many of the studies lack extensive serial sectioning to help identify the localisation of autophagy markers. Although, extensive evaluation of several autophagy markers in IPF did conclude that autophagy is reduced in IPF. One study did also include attempts to determine the localisation of staining,^37^ however, there were no H&E or Trichrome stains, which would be particularly helpful in not only determining the localisation of autophagy markers but also identifying their proximity to features of IPF, such as fibroblast foci.

## Autophagy regulates the pulmonary fibrosis process via different signaling

**Figure 2:**
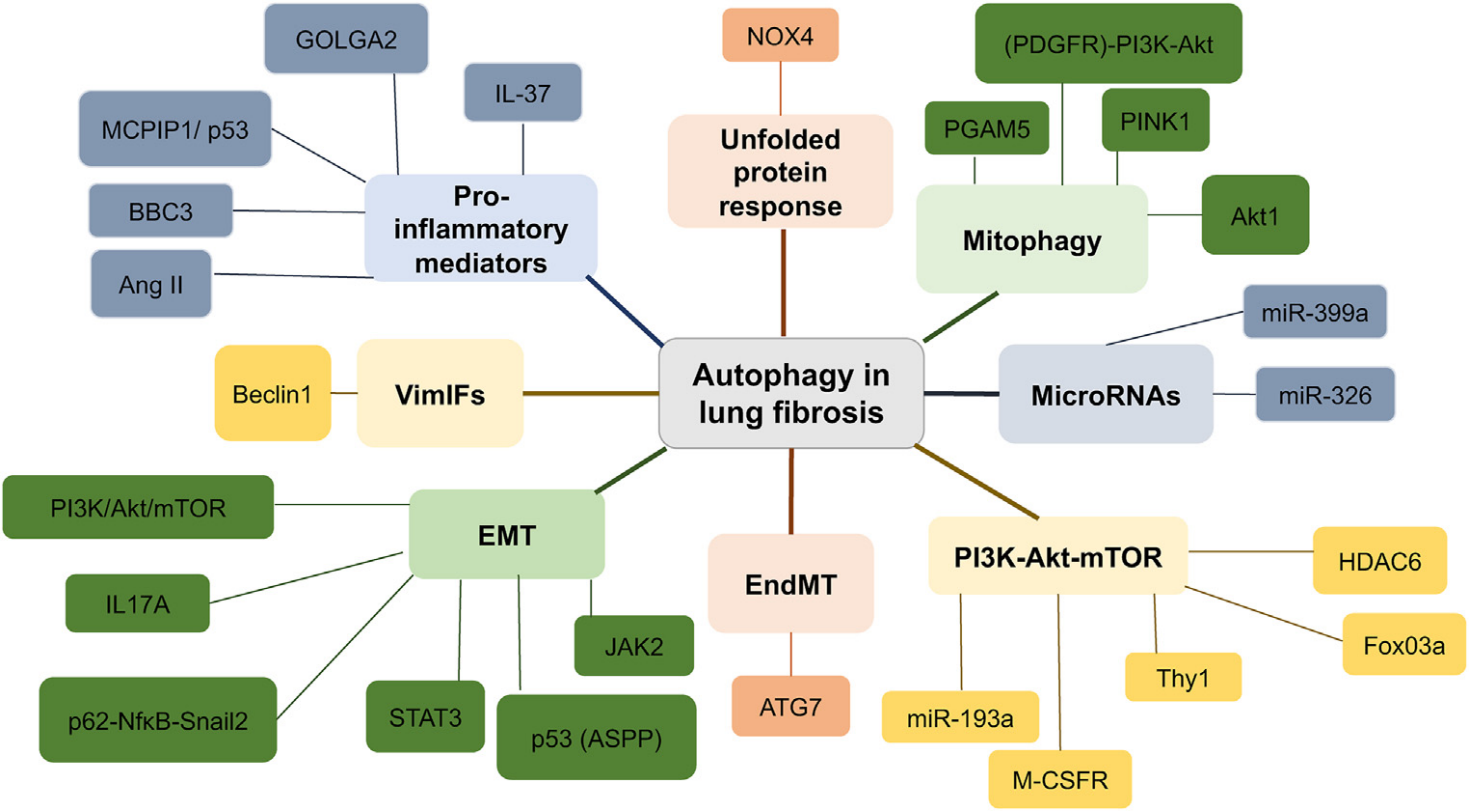
Summary of the role of autophagy in lung fibrosis. Each colour indicates the different processes and their mediators, which have been implicated with autophagy and lung fibrosis.

The role of autophagy in IPF is complex (Fig. 2), with some studies suggesting autophagy has an anti-fibrotic role (Table 2) and other studies finding a pro-fibrotic role for autophagy (Table 3). Determining whether autophagy has a protective effect in IPF, and further elucidating the signaling mechanisms which underpin its role in disease progression could be instrumental in the development of new drug targets.

**Table 2:** Anti-fibrotic roles of autophagy in IPF.

Anti-fibrotic roles of autophagy in lung fibrosis	Reference
Autophagy inhibition can induce EMT in ATII cells via the p62/SQSTM1-NFκB-Snail2 pathway. However this does not appear to drive fibrosis, instead secreted factors from autophagy-inhibited ATII cells promote fibrogenesis and this can be attenuated by depletion of Snail2.	Hill et al.^44^
Reduced levels of autophagy may be responsible for increased senescence and myofibroblast differentiation. Autophagy inhibition induces myofibroblast differentiation.	Araya et al (2013)^37^
IPF lung tissue shows reduced autophagy. *In vitro,* TGF-β inhibits autophagy in lung fibroblasts via mTORC1. Inducing autophagy in fibroblasts by rapamycin decreases α-SMA and fibronectin expression.	Patel et al (2012)^38^
ANXA2 direct binding target of BLM, binding stops TFEB induced autophagic flux and this can induce pulmonary fibrosis.	Wang et al (2018a)^47^
*Atg4b*-deficient mice can exacerbate BLM induced fibrosis. It was associated with an increase in neutrophil infiltration and changes in pro-inflammatory cytokines. Increased epithelial apoptosis. By 28 days post-BLM, extensive fibrosis as observed in *Atg4b*-deficient mice.	Cabrera et al (2015)^48^
STAT2 and JAK3 induced in IPF. Phosphorylation of both induces EMT in ATII cells and FMT in fibroblasts. Inhibition of STAT2 and JAK3 simultaneously resulted in an increase in autophagy, reduced fibroblast migration and senescence.	Milara et al (2018)^64^
Inhibition of VimIFs reduces the invasiveness of fibroblasts and can protect against murine-BLM induced fibrosis. Treatment with VimIF inhibitor increased autophagy and invasiveness of fibroblasts was reduced in the murine model, 3D organoids and IPF-derived pulmospheres.	Surolia et al (2019)^72^
Activation of autophagy in MRC5 cells, via PI3K/AKT/mTOR, protects from TGF-β induced fibrosis.	He et al (2020a)^51^
Autophagy inhibited after LPS challenge in mouse lung fibroblasts together with PI3K-Akt-mTOR pathway activation. LPS promotes lung fibroblast proliferation by autophagy inhibition via the PI3K-Akt-mTOR pathway.	Xie et al (2019)^92^
PQ induced PI3K/Akt/mTOR and Hh via miR-193a, together with an inhibition of autophagy; this increases fibrosis.	Liu et al (2019)^86^
S1PL increased in IPF. TGF-β can increase its expression. Overexpression of S1PL reduces TGF-β- and S1PL-induced differentiation via expression of LC3 and Beclin1.	Shuang Huang et al (2015)^56^
eEF2K increased in fibroblasts. eEF2K controls ECM deposition via p38 MAPK. Inhibition of eEF2K suppresses autophagy in fibroblasts treated with TGF-β.	Wang et al (2018b).^57^
Impaired autophagy was observed in BLM-treated mice, IL-17A Ab-treated mice had increased autophagy, resolved fibrosis.	Mi et al (2011)^70^
*In vitro*, knockdown of *ATG7* in endothelial cells (ECs) promotes endothelial–mesenchymal transition (EndMT). It also promotes TGF-β signaling and pro-fibrotic genes. *ATG7* EC-specific knockout mice demonstrates increased susceptibility to BLM-induced fibrosis.	Singh et al (2015)^73^
Overexpression of TFEB reduces lysosomal dysfunctional, increasing autophagy flux in alveolar macrophages, preventing fibrosis.	He et al (2020b)^51^
Autophagy is inhibited by SiNPs in ATII cells. Rapamycin treatment in mice induces autophagy and protects AECs from apoptosis to reduce SiNP-induced fibrosis.	Zhao et al (2019)^53^
*Lc3B* KO mice are more susceptible to BLM-induced fibrosis, epithelial cell apoptosis and elevated ER stress.	Kesireddy et al (2019)^55^
Leptin augments TGF-β1-induced EMT; this is mediated by inhibition of autophagy via the PI3K/Akt/mTOR pathway.	Gui et al (2018)^69^
Fibrosis related to reduced miR-449a expression. Overexpression of miR-449a reduces lung fibrosis by upregulating autophagy. Bcl2 found to be a target of miR-449a.	Han et al (2016)^83^
Reduced levels of miR-326 have also been reported in SiO_2_-murine models. Increased expression of miR-326 in SiO_2_-induced fibrosis, increases autophagy in fibroblasts and reduced fibrosis by downregulating both polypyrimidine tract-binding protein (PTBP1) and tumour necrosis factor superfamily 14 (TNFSF14).	Xu et al (2019)^84^
Activated Akt can induce collagen production in the BLM-murine model. A transgenic mouse model that constitutively expresses the active form of Akt (myristoylated AKT), also demonstrated reduced autophagy.	Dakhlallah et al (2019)^89^
FoxO3a, a direct target of Akt, has low expression in IPF fibroblasts. Reduced autophagy, via FoxO3a, contributes to fibrogenesis.	Im et al (2015)^79^
Histone deacetylase 6 (HDAC6) expression is reduced in IPF lungs. Inhibition of HDAC6 (with Tubastatin), reduces TGF-β induced collagen expression; via reduced p-Akt, autophagy and regulation of HIF-1α-VEGF. HDAC6 inhibition by Tubastatin reduces fibrosis via TGF-β-PI3K-Akt.	Saito et al (2017)^90^
Reduced levels of autophagy induction in IPF fibroblasts compared to young- and age-matched- normal fibroblasts. Aged IPF fibroblasts exhibit reduced starvation-induced autophagy, regulated via mTOR. IPF fibroblasts display mTOR activation, which contributes to apoptosis resistance. Inhibition of mTOR stimulates starvation-induced autophagy in young and old, but not IPF fibroblasts.	Romero et al (2016)^77^
BLM-treatment in mice displays activation of TGF-β and AKT/mTOR pathways. Younger-mice exposed to BLM exhibited more LC3 punctate. TGF-β1 inhibits autophagy and mitochondrial recycling in fibroblasts during FMT.	Sosulski *et al* (2015)^91^
LPS-induced autophagy-inhibition in lung fibroblasts, concomitantly with PI3K-Akt-mTOR activation; by reducing thymocyte differentiation antigen-1 (Thy-1) expression and increase in integrin b3 (Itgb3) expression.	Wan et al (2019)^93^
Elevated autophagy results in fibroblast senescence and inhibition of FMT via mTOR complex 2 (mTORC2).	Bernard et al (2020)^94^
Ang-(1–7) reduces smoking-induced fibrosis by activating autophagy and reducing NOX4-dependent ROS.	Pan et al (2018)^54^
In the BLM-induced murine model of fibrosis activated toll-like receptor 4 (TLR4) improved fibrosis and lung function, Inhibition of TLR4 abolished them. Increased autophagy, reversed the effect of TLR4 leading to reduced fibrosis; whereas autophagy inhibition reverses the anti-fibrotic roles of TLR4.	Yang et al (2012b)^52^
IL17A was shown to inhibit the phosphorylation of B-cell CLL/lymphoma 2 (BCL2). IL17A regulates the phosphorylation of BCL2 via the IL17A-PI3K-GSK3B-BCL2 signaling pathway.	Liu et al (2013a)^71^
In primary lung fibroblasts, TGF-β induced autophagy both ECM accumulation and UPR were attenuated with Baf-A1 (autophagy inhibitor).	Ghavami et al (2018)^102^
IL-37 reduced in IPF patients. IL-37 shown to reduce fibrosis by attenuating TGF-β1 signaling and inducing autophagy.	Kim et al (2019)^98^
In the BLM-mouse model, autophagy activation reduces Ang II-induced activation of NLPR3 by reducing ROS and mitochondrial dysfunction. Autophagy reduces fibrosis via NLRP activation, which is induced by Ang II-mediated ROS.	Meng et al (2019)^99^
Rapamycin-treated IPF fibroblasts modified starvation-induced autophagy and apoptosis. mTORC may contribute to the resistance of cell death.	Romero et al (2016)^77^
IPF-derived fibroblasts are resistant to type I collagen matrix-induced cell death. IPF fibroblasts have low levels of autophagic activity on polymerised collagen; aberrant PTEN-Akt signaling allows IPF fibroblasts to maintain their phenotype on collagen by suppressing autophagy.	Nho et al (2014)^78^
FoxO3a was found to mediate Akt resulting in autophagy suppression. Autophagy inhibition enhanced IPF fibroblast viability. Inhibition of miR-96 resulted in an increase in FOXO3a mRNA and protein levels, attenuating IPF fibroblasts proliferation and promoting cell death.	Nho et al (2014)^78^ Im et al (2015)^79^
*TOLLIP* protects bronchial epithelial cells from BLM-induced apoptosis by reducing mtROS and upregulating autophagy.	Li et al (2020)^80^

**Table 3:** Pro-fibrosis role of autophagy in lung fibrosis.

Pro-fibrostic roles of autophagy in lung fibrosis	Reference
SiO2-induced macrophage autophagy promoted proliferation and migration of fibroblasts.	Liu et al (2016, 2017)^82^^,^^111^
Autophagy upregulated in SiO_2_ induced lung fibrosis. In lung fibroblasts, SiO_2_ downregulated circRNA-012091 and induced up-regulation of downstream PPP1R13B. PPP1R13B regulates migration and proliferation of fibroblasts via ER stress and autophagy.	Cheng et al (2019)^65^
Serum starved fibroblasts with autophagy induced increased myofibroblast markers.	Bernard et al (2014)^88^
Inducing autophagy by deletion of Golgin A2 (GOLGA2), induced lung fibrosis.	Park et al (2018)^97^
Azithromycin has enhanced effects on lung fibroblasts from idiopathic pulmonary fibrosis (IPF) patients compared to controls, mediated by autophagy.	Krempaska et al (2020)^76^
Alveolar macrophages from *Park2*^−/−^ mice demonstrated increased apoptosis compared to BLM-injured WT mice	Larson-Casey et al (2016)^81^

Bleomycin (BLM) is widely used in animal models of IPF; clinically it is an anticancer drug which causes DNA strand breaks directly and is also known to cause fibrosis.^47^^,^^50^ Annexin A2 (ANXA2) has been identified as a direct target of BLM. *ANXA2*^*E*139*A*^ mutation in A549 cells prevents BLM binding and activates transcription factor EB (TFEB), a master regulator of the autophagy-lysosomal pathway, causing significant induction of autophagy. IPF patients had lower TFEB and LC3B levels than controls, whilst activation of TFEB increases autophagic-flux after BLM treatment, inhibiting apoptosis and proliferation of epithelial cells, thus reducing fibrosis.^47^ Further to this study, the overexpression of *TFEB* reduced lysosomal dysfunctional, increasing autophagy flux in alveolar macrophages thereby preventing fibrosis. It also reduced levels of inflammatory cytokines. Autophagy inhibition, together with *TFEB* knockdown was able to reverse these changes.^51^ These results suggest the importance of autophagy activation as a potential therapeutic target in IPF, specifically TFEB and the potential roles in lysosomal dysfunction.

In the BLM-induced murine model of fibrosis, activated toll-like receptor 4 (TLR4), is important in the regulation of innate immunity, improved fibrosis and lung function. Increased autophagy induced by rapamycin reversed the effects of TLR4, leading to reduced fibrosis; whereas autophagy inhibitor, 3-methyladenine (3-MA), exacerbated the fibrotic effects of TLR4, resulting in increased fibrosis and increased animal death.^52^ TLR4 is critical for mediating immunity and is key for attenuation of fibrosis and could be utilised in treatment; suggesting immunostimulants which utilise autophagy rather than immunosuppressants which suppress autophagy could be utilised.^52^

Dysfunctional autophagy and subsequent apoptosis in ATII cells have been demonstrated to have a role in silica nanoparticle (SiNP)-induced fibrosis. Autophagy is inhibited by SiNPs in ATII cells, through the impairment of lysosomal degradation through alterations in lysosomal acidification. Rapamycin treatment in mice induces autophagy and protects ATII cells from apoptosis, reducing SiNP-induced fibrosis.^53^ In smoking-induced lung fibrosis, Angiotensin (1–7) reduced smoking-induced fibrosis by activating autophagy and reducing NOX4-dependent ROS. Autophagy inhibitors, 3-MA and Baf-A1, were able to attenuate the protective effects of Ang-(1–7)^54^. Further, *LC3B* KO mice were shown to have increased susceptibility to BLM-induced fibrosis; this also resulted in epithelial cell apoptosis and increased ER stress.^55^

Sphingosine-1-phosphate (S1P) signaling is important in the pathogenesis of IPF. Expression of S1P lyase (S1PL) is upregulated in both fibrotic tissue and primary lung fibroblasts compared to controls, as well as in BLM-treated mice. TGF-β was found to increase the expression of S1P, through the binding and activation of Smad3 transcription factor to the Sgpl1 promoter. *In vitro*, over-expression of S1PL reduced TGF-β- and S1P-induced fibroblast differentiation via LC3 and Beclin. *S1PL*^*-/*+^ BLM-treated mice displayed increased fibrosis. Further elucidation of the mechanisms underlying these processes could be beneficial in the development of drug treatment; either by targeting S1P directly or via interactions with components in autophagy signaling.^56^ Elongation factor-2 kinase (eEF2K) negatively regulates protein synthesis and has been shown to modulate fibroblast-myofibroblast transition (FMT). eEF2K inhibition augments TGF-β-induced FMT and resistance to apoptosis. Further, inhibiting eEF2K induces FMT, reducing myofibroblast autophagy through p38 MAPK signaling.^57^

### Epithelial-mesenchymal transition and endothelial–mesenchymal transition

Epithelial-mesenchymal transition (EMT) has been identified as a key process in the pathogenesis of IPF.^2^^,^^4^^,^^44^^,^^58^ EMT is a reversible, biological process where epithelial lose cell polarity, adherens and tight junctions in favour of a mesenchymal phenotype. It has been implicated in cancer, development, and fibrosis, and can cause an increase in the migratory and invasive ability of cells.^59^, ^60^, ^61^ Autophagy and EMT have a complicated relationship that appears to be both context- and tissue-dependent. A number of recent studies have begun to elucidate underlying mechanisms that drive autophagy-driven EMT. Recent studies also suggest that the role of EMT may go beyond a direct phenotypic conversion, instead cells undergoing EMT may secrete factors that can induce fibrosis without directly contributing to the pool of fibroblasts themselves.^58^^,^^62^^,^^63^

The roles of autophagy and EMT in lung fibrosis have been reported by some groups. Janus Kinase 2 (JAK2) and signal transducer and activator of transcription 3 (STAT3) are both activated in IPF and phosphorylation of both induces EMT in ATII cells and fibroblast-myofibroblast (FMT) in the lung. Inhibition of both reduces fibroblast migration, attenuates fibroblast senescence, and increases autophagy.^64^ Protein phosphatase 1 regulatory subunit 13B (PPP1R13B) is a member of the apoptosis-stimulating proteins of the p53 (ASPP) family. In lung fibroblasts, SiO_2_ downregulated circRNA-012091 and induced upregulation of downstream PPP1R13B. It is thought that PPP1R13B regulates circ-012,091 to promote both migration and proliferation of fibroblasts by ER stress and autophagy.^65^

TGF-β1-induced EMT in IPF is controversial, with some studies demonstrating that TGF-β can induce characteristics of EMT in some alveolar epithelial cell lines (such as A549 cells, which harbor a KRAS mutation).^66^, ^67^, ^68^ Whilst other studies in primary ATII cells found that TGF-β was unable to induce EMT.^58^ Leptin, a protein product of the obesity gene, augments TGF-β1-induced EMT. These effects were mediated by inhibition of autophagy via the PI3K/Akt/mTOR pathway.^69^

IL17A is increased in IPF; it has been shown to induce EMT and is responsible for the secretion of the synthesis and secretion of collagen in a TGF-β1-dependent manner. Impaired autophagy was observed in BLM-treated mice, whilst IL-17A Ab-treated mice had increased autophagy.^70^ IL-17A was shown to inhibit phosphorylation of B-cell CLL/lymphoma 2 (BCL2), a protein involved in the regulation of apoptosis, in lung epithelial cells, subsequently preventing the degradation of BCL2. As a result, autophagy was reduced due to increased interaction of BCL2 and BECN1. IL-17A regulates the phosphorylation of BCL2 via the IL17A-PI3K-GSK3B-BCL2 signaling pathway.^71^ IL-17A was shown to inhibit phosphorylation of BCL2, a protein involved in the regulation of apoptosis in lung cells, subsequently preventing the degradation of BCL2; as a result, autophagy was reduced due to increased interaction of BCL2 and BECN1.^71^

Increased invasion is a characteristic of IPF fibroblasts and vimentin can regulate this through the increased assembly of vimentin intermediate filaments (VimIFs). VimIFs have been linked with proteins involved in the regulation of autophagy. In IPF fibroblasts, VimIFs form a complex with Beclin1 which inhibits autophagy. Withaferin A, a plant-based alkaloid that binds to vimentin at Cys328, which is crucial for remodeling in cells, can inhibit VimIF assembly. Treating IPF fibroblasts with Withaferin A can diminish the interaction between Beclin1 and VimIFs. It also protects the lungs from fibrosis via increased autophagy in murine-BLM models.^72^

Endothelial-mesenchymal transition (EndMT) may be important in the pathogenesis of IPF. A recent study demonstrated that loss of autophagy gene *ATG7* could induce EndMT *in vitro*. There was a loss of endothelial cell (EC) architecture, as well as an increase in mesenchymal markers accompanied by a loss in endothelial markers. *In vivo* EC-specific knockout of *Atg7* in mice augmented fibrosis and collagen accumulation.^73^ These findings suggest that inhibition of autophagy induces EndMT and autophagy could be a potential target in fibrosis.

In a similar manner to the findings presented by Singh et al*,* ^73^ we recently reported,^44^ in IPF that autophagy inhibition was able to induce EMT in alveolar epithelial cells. We demonstrated that both chemical inhibition (with autophagy inhibitors bafilomycin or hydroxychloroquine), or genetic inhibition (RNAi against *ATG5*), was sufficient to induce EMT in ATII and A549 cells. These results were confirmed by a number of biochemical assays, as well as invasion and migration assays.^44^ We determined that EMT in ATII cells was via the p62-NF-κB-Snail2 pathway, in a similar manner to previously reported in malignancy.^74^ However, these autophagy-inhibited alveolar cells did not produce significant amounts of collagens; suggesting that although they had undergone EMT they had not converted to myofibroblasts, which are key drivers in the pathogenesis of IPF. We instead found that secreted factors from these cells mediated fibrogenesis. Further, using conditioned media from ATII cells which had the inhibition of both *ATG5* and *SNAI2* (Snail2), was sufficient to attenuate α-SMA in IPF fibroblasts cells; suggesting this process was driven by Snail2 (*SNAI2*).^44^

### Apoptosis

Autophagy and apoptosis are closely related processes; autophagy can prevent cells from undergoing apoptosis.^75^ Several studies have found links between apoptosis and autophagy in pulmonary fibrosis. Understanding the links between these processes could be harnessed to target new treatments. Azithromycin attenuated fibrosis and enhanced early apoptosis in IPF fibroblasts compared to control fibroblasts. Azithromycin also impaired autophagic flux in IPF fibroblasts. Azithromycin has both anti-fibrotic and pro-apoptotic effects on primary fibroblasts which may be mediated by autophagy.^76^ Another study found by treating IPF fibroblasts with rapamycin modified starvation-induced autophagy as well as apoptosis; it is thought that the activation of mTORC may contribute to the resistance of cell death in IPF fibroblasts.^77^

IPF-derived fibroblasts are resistant to type I collagen matrix-induced cell death. IPF fibroblasts have low levels of autophagic activity on polymerised collagen; aberrant PTEN-Akt signaling allows IPF fibroblasts to maintain their phenotype on the collagen by suppressing autophagy. Inhibition of autophagy in IPF fibroblasts over-expressing PTEN or dominant negative Akt increases IPF fibroblast cell death. In IPF lung tissue LC3-II is low, whilst mTOR levels are high within the fibroblastic foci. These findings suggest dysregulated autophagy may be important in preserving IPF fibroblasts phenotype in a collagen-rich environment.^78^ FoxO3a was found to mediate Akt resulting in autophagy suppression. FoxO3a is involved in the transcriptional activity of autophagy and is a direct target of Akt.^79^ The inhibition of autophagy enhanced viability in IPF fibroblasts compared to control; low FoxO3 reduces autophagic activity by the transcriptional suppression of LC3B in IPF fibroblasts on collagen.^79^ MicroRNA-96 (miR-96) binds to the 3′-UTR region of FOXO3a mRNA and inhibits its function. MiR-96 levels are increased in IPF fibroblasts whilst FOXO3a levels are reduced in IPF fibroblasts when cultured on collagen. Inhibition of miR-96 resulted in an increase in FOXO3a mRNA and protein levels, attenuating IPF fibroblasts proliferation and promoting cell death.^78^

Mitophagy has been implicated in IPF, it may exert its effects by modulating apoptosis. ER stress modulates mitochondrial function in ATII cells via the down regulation of PINK1 leading to an increase in apoptotic mitochondrial responses.^24^ In IPF, Toll interacting protein (*TOLLIP*) protects bronchial epithelial cells from BLM-induced apoptosis and these effects are mediated by reducing mtROS and up-regulating autophagy^80^; TOLLIP was significantly reduced in IPF lungs compared to control.^80^ Akt1-mediated mitophagy has also been shown to contribute to macrophage apoptosis resistance in alveolar cells.^81^ Mitophagy is increased in alveolar macrophages. Mitophagy can be induced by ROS; Akt1 increases mitochondrial ROS. Akt1 mediates mitochondrial ROS in turn inducing autophagy in IPF alveolar cells. Macrophages were found to be resistant to apoptosis. Alveolar macrophages from *Park2*^−/−^ mice demonstrated increased apoptosis compared to BLM-injured WT mice.^81^ SiO_2_-induced macrophage autophagy which was associated with augmented expression of monocyte chemotactic protein-1-induced protein 1 (MCPIP1). Autophagy promoted apoptosis. Autophagy was induced in macrophages following silica exposure. SiO_2_ induced MCPIP1 expression in macrophages which acted via p53 to mediate autophagy. Autophagy was both responsible for the activation of macrophages and critical for macrophage apoptosis in response to silica; macrophage autophagy was mediated by MCPIP1. Silicosis patients were found to have increased autophagy, apoptosis and activation in macrophages.^82^

### MicroRNAs

Altered levels of microRNAs have been shown to contribute to the pathogenesis of lung fibrosis, however, recent studies have also linked this to autophagy. Silica induced-fibrosis in the murine model and TGF-β treated fibroblasts both show miR-449a to be reduced. Overexpressing miR-449a reduced lung fibrosis both *in vitro* and *in vivo* by upregulating autophagy, and Bcl2 was determined to be the autophagy-related target of miR-449a.^83^ Reduced levels of miR-326 have also been reported in both silica induced-fibrosis murine models and in several *in vitro* studies (lung epithelial cells and fibroblasts). Increased expression of miR-326, led to increased autophagy in fibroblasts and reduced fibrosis by downregulating both polypyrimidine tract-binding protein (PTBP1) and tumor necrosis factor superfamily 14 (TNFSF14).^84^

### PI3K-Akt-mTOR pathway

In BLM-induced fibrosis, autophagy was shown to be activated; LC3 expression was increased by day 28, whilst p62/SQSTM1 was reduced. BLM-treated *Atg4b* deficient mice displayed exacerbated fibrosis and cellular apoptosis.^48^ This suggests Atg4b may have a protective mechanism — an upregulation of Atg4b in old fibroblasts which is involved in the lipidation of LC3, may have a role in the reduction of fibrosis.^77^^,^^85^ BLM-treated mice also exhibited increased expression of p-S6, a downstream effector of mTOR. IPF lung tissue also showed increased staining of p-S6 suggesting mTOR activation. *In vitro* studies, demonstrated that fibroblasts treated with TGF-β increased mTOR expression. Conditional knockdown of Tsc1 (which regulates mTOR) in AECs in BLM mice, augments fibrosis, which was attenuated with rapamycin treatment. This could then be reversed by autophagy inhibitor chloroquine.^49^ Long term paraquat (PQ) treatment increased levels of ROS, resulting in increased mTOR activity, which led to autophagy inhibition and finally augmented fibrosis suggesting that PQ-induced fibrosis reduced the activity of miR-193a.^86^

TGF-β is fundamental in the pathogenesis of IPF, and PI3K/AKT/mTOR are downstream of this. TGF-β also has roles in the regulation of autophagy. However, the exact mechanisms of this in IPF are not fully understood. A recent study demonstrated PI3K/AKT/mTOR pathway activation upon TGF-β treatment of fibroblasts. Treatment with a natural flavonoid isoliquiritigenin (ISL) inhibited both pathway activation and phosphorylation of these. It also activated autophagy and decreased a number of fibrotic markers, suggesting that PI3K/AKT/mTOR may be key in regulating autophagy and fibrosis.^87^ TGF-β has been previously shown to inhibit autophagy in primary lung cells, via mTORC1. In this study, inhibition of autophagy by siRNA caused an increase in α-SMA.^38^ Conversely, other studies have shown that fibroblasts with serum starvation induced-autophagy demonstrated induction of several myofibroblast markers including α -SMA.^88^

In macrophages, macrophage colony-stimulating factor (M-CSF-receptor) activates the PI3K-AKT pathway in macrophages. Activated Akt can induce collagen production in the BLM-murine model. A transgenic mouse model that constitutively expresses the active form of Akt (myristoylated AKT), also demonstrated reduced autophagy.^89^ FoxO3a, a direct target of Akt, has low expression in IPF fibroblasts and in turn, reduces LC3B expression. Whereas healthy fibroblasts displayed high FoxO3a and LC3B expression. FoxO3a binds to the promoter region of LC3B, transcriptionally activating it. Autophagy inhibition in control fibroblasts increased collagen matrix induced cell death whilst in IPF fibroblasts it resulted in increased viability. When FoxO3 expression is low it reduces autophagic activity, subsequently suppressing LC3B in IPF fibroblasts; this suggests that reduces autophagy, via FoxO3a contributes to fibrogenesis.^79^

Histone deacetylase 6 (HDAC6) expression is reduced in IPF lungs. Inhibition of HDAC6 (with Tubastatin), reduces TGF-β induced collagen expression through reduced p-Akt, autophagy and regulation of HIF-1α-VEGF. Although the study only evaluated autophagy with LC3 levels, so further investigation into this with other markers would be beneficial. WT mice treated with Tubastatin are less susceptible to BLM-fibrosis whereas *HDAC6* KO mice are not. Suggesting HDAC6 inhibition by Tubastatin reduces fibrosis via TGF-β-PI3K-Akt, independent of HCAD6.^90^

Given that IPF is an ageing-associated disease and dysfunctional autophagy is often observed with ageing, understanding the mechanisms linking these processes is key. In fibroblasts, ageing has been associated with reduced levels of autophagy induction in IPF fibroblasts compared to young- and age-matched-normal fibroblasts. Aged IPF fibroblasts have reduced starvation-induced autophagy and this is regulated via mTOR. IPF fibroblasts demonstrate persistent mTOR activation, which has been shown to contribute to apoptosis resistance. Inhibition of mTOR can attenuate the effects of starvation-induced autophagy in both old- and IPF-fibroblasts.^77^ Murine models of fibrosis suggest that susceptibility to fibrosis in ageing correlates with reduced autophagy. BLM-treatment in mice resulted in activation of TGF-β and AKT/mTOR pathways. Younger mice (2 months *vs*. 14 and 22 months) exposed to BLM exhibited more LC3 punctate. TGF-β1 inhibits autophagy and mitochondrial recycling in fibroblasts during FMT. These findings suggest that reduced autophagy may be key in the pathogenesis of age-related lung conditions.^91^

Similarly, autophagy was inhibited in mouse lung fibroblasts after lipopolysaccharide (LPS) challenge and was accompanied by PI3K-Akt-mTOR pathway activation. Treatment with pathway (mTOR or PI3K-Akt) inhibitors could reverse this effect. Further, autophagy inhibition could promote fibroblast proliferation and mTOR inhibition (by rapamycin) could reverse this.^92^ A further study demonstrated LPS-induced autophagy inhibition in lung fibroblasts, together with PI3K-Akt-mTOR activation via a reduction in thymocyte differentiation antigen-1 (Thy-1) expression and an increase in integrin b3 (Itgb3) expression. LPS reduces binding of Thy-1 to Itgb3. Findings were demonstrated in MRC5 cells and in a mouse model of LPS-induced pulmonary fibrosis.^93^ These findings further confirmed the importance of both the PI3K-Akt-mTOR pathway and autophagy in the pathogenesis in IPF, but also give insight into potential novel therapeutic targets in these pathways.

Both autophagy and senescence contribute to fibrogenesis. A recent study has shown that persistent upregulation of autophagy results in fibroblast senescence and inhibition of FMT via mTOR complex 2 (mTORC2). Fibroblasts with serum starvation-induced autophagy displayed an increase in senescence; senescence and FMT were shown to be mutually exclusive. Inhibition of senescence increased myofibroblast differentiation. mTORC2 activation controls the expression of senescence markers and myofibroblast markers via signaling pathways independent of mTORC1.^94^

### Pro-inflammatory mediators

A mechanism that may augment fibrosis in the lung, is pro-inflammatory mediators secreted by macrophages. These macrophages may exacerbate fibrosis further by activating autophagy. Pulmonary exposure to silica particles can lead to the development of silicosis. This is characterised by inflammation, fibrosis and reduced lung function.^95^ Recent studies have shown SiO_2_ induced macrophage activation and apoptosis, as well as levels of autophagy by BCL2 binding component 3 (BBC3). These effects were blocked by autophagy inhibition (3-MA) and enhanced by autophagy induction with rapamycin. Conditioned media (CM) from macrophages treated with SiO_2_ led to increased proliferation and migration of fibroblasts. These findings were confirmed in a *Bbc3* knockout mouse model, which exhibited reduced levels of both autophagy and fibrosis.^96^ Similarly, SiO_2_-induced macrophage autophagy, together with an increase in monocyte chemotactic protein-1-induced-protein 1 (MCPIP1), promoted apoptosis. Macrophages promoted proliferation and migration of fibroblast via MCPIP1/p53 pathway.^82^ Another study has shown that deletion of GOLGA2, which encodes for a cis-Golgi protein, can induce autophagy and this results in fibrosis together with an increase in alveolar macrophages.^97^

Pro-inflammatory cytokine IL-37 is reduced in IPF compared to healthy controls, this is confirmed in AECs and macrophages, as well as lungs of mice exposed to BLM. IL-37 reduces the expression of fibrotic mediators as well as inhibiting cell death induced by oxidative cell death. Further, IL-37 can inhibit fibroblast proliferation through the inhibition of TGF-β1 signaling. IL-37 enhanced both ATG7 and Beclin-1 expression in lung fibroblasts, and also attenuated lung inflammation and fibrosis by activating autophagy in BLM-treated mice.^98^ Lung fibrosis is exacerbated by Angiotensin (Ang) II via NLR family pyrin domain containing 3 (NLRP3) pathways. In the BLM-mouse model, autophagy activation was shown to reduce Ang II-induced activation of NLPR3 by reducing ROS and subsequent mitochondrial dysfunction. Autophagy reduces fibrosis via NLRP activation, which is induced by Ang II-mediated ROS.^99^

### Unfolded protein response

Increasing attention has been given to the processes of unfolded protein response (UPR) and autophagy. Both of these biological processes are hallmarks of ageing and have been identified in the pathogenesis of IPF. UPR is initiated when misfolded or unfolded proteins are in abundance; in turn signaling from the ER to the nucleus maintaining homeostasis.^100^

TGF-β induced NADPH oxidase 4 (NOX4) expression and myofibroblast differentiation, could both be attenuated by azithromycin (AZM) treatment of lung fibroblasts. AZM-induced NOX4 reduction could be restored with a proteasome inhibitor. AZM inhibited autophagy, and this was associated with ubiquitination of NOX4 by increased STUB1 (STIP1 homology and U-box containing protein 1) levels, an E3 ubiquitin ligase. AZM also resulted in enhanced UPR which was linked with an increase in proteasome activity. BLM-induced fibrosis was reduced in severity by AZM, whilst NOX4 protein levels were reduced and proteasome activation was increased. These suggest AZM may be a possible therapy for fibrosis, by suppressing NOX4 and promoting proteasomal degradation leading to inhibition of TGF-β-induced fibrogenesis.^101^ A further study examining the anti-fibrotic effects of AZM confirmed it reduced expression of pro-fibrotic genes after TGF-β in both control and IPF fibroblasts. AZM was shown to have increased anti-fibrotic effects on a number of fibrotic and pro-apoptotic markers in IPF fibroblasts compared to controls, it is thought that impaired lysosomal function may contribute to these effects. Given these findings, there may be potential for the use of AZM as an anti-fibrotic treatments in IPF.^76^

Another study demonstrated in primary fibroblasts, that TGF-β initiated both autophagy, and UPR and ECM accumulation. This could be attenuated upon Baf-A1 treatment.^102^ Conversely, TGF-β has been previously shown to inhibit autophagy in fibroblasts and autophagy inhibition increased α-SMA expression.^38^ The differences between these studies could be as a result of the different time points used for TGF-β treatment (48 h ^38^ or 120 h ^102^), or that the treatment with Baf-A1 had some off-target effects, further studies using genetic knockdown would help validate their findings.

### Mitophagy

Mitophagy is a selective form of autophagy that targets dysfunctional mitochondria for degradation by autophagosomes.^103^ This is an important process for maintaining cellular homeostasis. Mitophagy can be induced by mitochondrial oxidative stress.^81^ A number of lung conditions, including IPF, have reported dysfunctional mitophagy. In a similar manner to (macro) autophagy, mitophagy has been described to have dual-roles, with some recent studies suggesting reduced mitophagy can augment fibrosis. ROS is in the pathogenesis of IPF and it is thought ROS generated from mitochondria may promote fibrosis.^104^ Further to this, it has been shown that TGF-β not only promotes ROS production^105^ but that latent TGF-β can be activated with oxidative stress.^106^

Akt1 can induce macrophage mtROS and also mitophagy; it also increased TGF-β1 expression. Mitophagy inhibition in Akt1-overexpressing macrophages can reverse the increase in TGF-β expression and fibroblast differentiation. Mice harboring conditional deletion of Akt1 in macrophages had increased mitophagy and macrophage apoptosis and were protected from fibrosis.^81^ *Park2*^*−/−*^ mice exhibited reduced mitophagy and increased FMT mediated by the activation of the platelet-derived growth factor receptor (PDGFR)-P13K-Akt pathway upon BLM-treatment.^107^

A recent study demonstrated a role for phosphoglycerate mutase family member 5 (PGAM5) in the pathogenesis of IPF. *PGAM5* knockout mice treated with BLM displayed significantly reduced fibrogenesis in the lung compared with control. *In vitro* studies further confirmed its role, showing *PGAM5* knockout in alveolar cells had reduced structural damage to pulmonary architecture and inflammatory changes. They further showed that PGAM5 impaired mitochondrial integrity (mitochondrial membrane potential, and mitochondrial depolarization, structural imaging) independent of mtROS-production (which is increased on BLM treatment). Loss of *PGAM5* induced mitophagy, and this improved mitochondrial homeostasis.^108^

PINK1 acts as a molecular sensor of damaged organelles during mitophagy functioning as a serine/threonine kinase containing a mitochondrial targeting sequence.^109^ The role of PINK1 in IPF is controversial. IPF patients have been demonstrated to have an accumulation of damaged mitochondria, and these have been associated with low PINK1 expression.^24^ Conversely, another study found increased PINK1 expression to be associated with the accumulation of damaged mitochondria.^110^ Studies *in vitro* and *in vivo* demonstrated that knockdown of *PINK1* resulted in dysfunctional mitochondria in ATII cells and defective mitophagy.^24^ Further, TGF-β1 induced dysfunction of mitochondria and increased PINK1 expression, these changes were reversed by ROS scavenging.^110^ Together these findings suggest that PINK1 may be a potential target for new treatments, as PINK1 has been shown to reduce epithelial cell death^110^ and subsequently could improve fibrosis.

## Conclusion

Autophagy has been widely implicated in a number of diseases and its impact on fibrosis is still being explored. Several studies have determined that autophagy is dysregulated in IPF lungs and this suggests that it could be an important area for further investigation. The effects of autophagy misfunction are wide-reaching; broadly it seems that autophagy may have a protective effect although further investigation is certainly required as it seems to be context-dependent. Given that the manipulation of autophagy has already been utilised for the treatment of cancers, this could be an exciting prospect in the treatment of fibrosis.

## Author contributions

Charlotte Hill conceptualised, wrote and edited the manuscript. Yihua Wang conceptualised, supervised and acquired funding for this project.

## Funding

This work was supported by the 10.13039/501100000265Medical Research Council (No. MR/S025480/1), the 10.13039/501100000691Academy of Medical Sciences/the 10.13039/100010269Wellcome Trust Springboard Award (No. SBF002\1038), 10.13039/501100008176Wessex Medical Trust and 10.13039/501100005188AAIR Charity. CH was supported by 10.13039/100012166Gerald Kerkut Charitable Trust and University of Southampton Central VC Scholarship Scheme.

## Declaration of competing interest

Authors declare no conflict of interests.
